# Socioeconomic Disparities in Maternity Care among Indian Adolescents, 1990–2006

**DOI:** 10.1371/journal.pone.0069094

**Published:** 2013-07-23

**Authors:** Chandan Kumar, Rajesh Kumar Rai, Prashant Kumar Singh, Lucky Singh

**Affiliations:** 1 Department of Humanities and Social Sciences, Indian Institute of Technology Roorkee (IITR), Roorkee, Uttarakhand, India; 2 Tata Institute of Social Sciences (TISS), Mumbai, India; 3 International Institute for Population Sciences (IIPS), Mumbai, India; 4 School of Health Systems Studies, Tata Institute of Social Sciences (TISS), Mumbai, India; Indiana University and Moi University, United States of America

## Abstract

**Background:**

India, with a population of more than 1.21 billion, has the highest maternal mortality in the world (estimated to be 56000 in 2010); and adolescent (aged 15–19) mortality shares 9% of total maternal deaths. Addressing the maternity care needs of adolescents may have considerable ramifications for achieving the Millennium Development Goal (MDG)–5. This paper assesses the socioeconomic differentials in accessing full antenatal care and professional attendance at delivery by adolescent mothers (aged 15–19) in India during 1990–2006.

**Methods and Findings:**

Data from three rounds of the National Family Health Survey of India conducted during 1992–93, 1998–99, and 2005–06 were analyzed. The Cochran-Armitage and Chi-squared test for linear and non-linear time trends were applied, respectively, to understand the trend in the proportion of adolescent mothers utilizing select maternity care services during 1990–2006. Using pooled multivariate logistic regression models, the probability of select maternal healthcare utilization among women by key socioeconomic characteristics was appraised. After adjusting for potential socio-demographic and economic characteristics, the likelihood of adolescents accessing full antenatal care increased by only 4% from 1990 to 2006. However, the probability of adolescent women availing themselves of professional attendance at delivery increased by 79% during the same period. The study also highlights the desolate disparities in maternity care services among adolescents across the most and the least favoured groups.

**Conclusion:**

Maternal care interventions in India need focused programs for rural, uneducated, poor adolescent women so that they can avail themselves of measures to delay child bearing, and for better antenatal consultation and delivery care in case of pregnancy. This study strongly advocates the promotion of a comprehensive ‘adolescent scheme’ along the lines of ‘Continuum of Maternal, Newborn and Child health Care’ to address the unmet need of reproductive and maternal healthcare services among adolescent women in India.

## Introduction

“If adults know child marriage is wrong, why do they allow it to happen?” a teenage girl asked in Bihar (India), where nearly 69% of the girls are married before age 18 [1]. According to Graca Machel, the first education minister of Mozambique, child marriage will hinder the “achievement of six of the eight Millennium Development Goals (MDG)”. Graça Machel and Desmond Tutu, the Archbishop Emeritus of Cape Town and a Nobel Peace Prize laureate, in an opinion column in the *Washington Post* wrote on August 1, 2012, about the current state of child marriage and urged United States legislators and the Obama administration to put an end to child marriage (marriage before age 18), to make it a priority issue and part of their foreign policy goals [1]. Global actors signed a consensus in 2000 as the Millennium Declaration to discourage adolescent fertility in order to reduce maternal mortality and to extend universal healthcare. It has been more than a decade since the declarations were signed, yet most of the developing countries are struggling to reduce high adolescent fertility [2]. According to the report on World Fertility Policies, 2011 [3], the adolescent birth rate (ABR, birth per 1000 women aged 15–19) of the world (estimates available for 172 countries) is 56 births per 1000 women; the estimates are 24 for the more developed regions and 105 for the least developed countries. Adolescent fertility and their maternity care is as global the concern as it is for India, although the context and underlying factors defer across countries.

Despite having policies to reduce adolescent fertility, the reduction of ABR in India (reported at 86 births per 1000 women aged 15–19) remains a grave concern [3]. Although, the country does not record the ABR as high as other African countries, but the total number of women aged 15–19 subject to adolescent fertility is relatively higher in India. While a much needed fertility decline has been experienced in India [4], the unacceptably high rate of early age at marriage (nearly 44.5% women aged 20–24 married before their 18th birthday) and low level of contraceptive use lead to early, frequent and unplanned pregnancies, which are often documented as emblematic causes of high mortality among young mothers [5], [6]. India, with a population of more than 1.21 billion has the highest maternal deaths in the world, estimated to be 56000 in 2010 [7]; and adolescent (aged 15–19) mortality shares 9% of total maternal deaths [8]. In India, marriage at a very young age is considered as the main reason for early pregnancy [5], compounded with poverty, low status of women, poor education and low level of contraceptive use [9]. The evidences suggest that the main reasons for adverse health consequences for teenage mothers are that these adolescents often lack experience and tend to be psychologically less mature and emotionally less stable, which leads to poor maternal and child health outcomes [10]. The early marriage pattern force young girls to leave school after marriage, particularly in rural areas in order to devote themselves full time to domestic work [11]. This ultimately restricts their ability to acquire knowledge about better health and thus threatens their health care. Moreover, a few circumstances such as poverty and single-parent household place girls at a higher risk of becoming teenage mothers [9].

A few recent studies document that the utilization of maternal and child healthcare services among adolescent women is far from satisfactory in India [12], [13]. Most cases of maternal morbidity and mortality can be prevented through scaling up the utilization of required maternal health care services. Poor utilization of maternity care is the outcome of the complex interaction of socioeconomic and demographic factors, which decides the accessibility, availability and affordability of health care services [14]–[16]. A growing body of literature suggests that the poor and uneducated women and those residing in rural areas, are susceptible to underutilize maternity care services [13], [17]–[19]. The development in utilization pattern of maternity care services with the dynamics of socioeconomic disparities among adolescent women in India is desirable to outline effective programmes in order to follow the fifth Millennium Development Goal (MDG-5), which aims to reduce the maternal mortality ratio by 75% between 1990 and 2015 [14]. Moreover, the government programmes such as *Janani Suraksha Yojana* (JSY) under the broad umbrella of the National Rural Health Mission (NRHM), provide financial support to pregnant mothers/women aged 19 years and above to promote late marriage [20]. Acknowledging the policy perspective as well, this is important to revisit the statistics that may warn us about the losses of overlooking the maternity needs of adolescent women.

This paper examines the extent of disparities in the use of maternity care services among adolescent women across key socioeconomic population groups in India over one and a half decades. In addition, the paper illustrates the unacceptable differences in the utilization of maternity care services by adolescent women belonging to two extreme ends of the persisting socioeconomic nexus in rural and urban areas. The term “socioeconomic nexus” frequently used throughout this paper refers to the interaction of select socioeconomic indicators such as education, social group/caste, and wealth quintile. Being at the disadvantageous end of these socioeconomic indicators (e.g. being illiterate, belonging to deprived social group/caste, and the lowest wealth quintile) is referred as the least favoured group, while possessing the most advantageous mix of these indicators corresponds to the most favoured group. Two components of maternal health care services are analyzed, that is, the adolescent women who received full antenatal care and had delivery assisted by health professionals.

## Methods

### Data

This study is based on three rounds of the National Family Health Survey (NFHS) data, which were canvassed during 1992–93 (NFHS-1), 1998–99 (NFHS-2), and 2005–06 (NFHS-3) in India [21]–[23]. These surveys used a multistage stratified sampling design. The NFHS is a standard large-scale survey in India, which provides nationally representative estimates on issues related to family welfare, maternal and child healthcare, and nutrition. Different rounds of NFHS collected information related to antenatal care and assistance during delivery (childbirth) for different reference periods. In NFHS-1 (1992–93), information was collected for the last three births to women in the four years preceding the date of survey. Similarly, in NFHS-2 (1998–99), information was collected for the last two births in the three years preceding the date of survey. However, in NFHS-3 (2005–06), information on antenatal care received by women was collected only for the last birth and the information on assistance during delivery was collected for the last three births in the five years preceding the date of survey. Considering these inconsistencies across the three surveys, the sample for this study was limited to the information for the last birth in the three years preceding the date of survey. Required information/data in states of Sikkim and Tripura (both the states are in the northeastern part of India) were missing in NFHS-1 and NFHS-2, respectively. Therefore, in order to retain consistency, samples from Sikkim and Tripura were excluded from the final analytic samples. The final analytic sample size for each survey is reported in **Appendix S1**. The individual response rate for women was 96.1% in NFHS-1, 95.5% in NFHS-2, and 94.5% in NFHS-3. Appropriate sample weights were used taking into account the survey design. The details of the sampling weights as well as extensive information on survey design, data collection, and management procedures are described in the NFHS reports of the respective rounds [21]–[23].

### Ethics Statement

The NFHS was conducted under the scientific and administrative supervision of the International Institute for Population Sciences (IIPS), Mumbai, India. The institute (IIPS) conducted an independent ethics review of NFHS protocol. Data collection procedures were also approved by the ORC Macro institutional review board. This study is based on the NFHS data, which is available in the public domain to use with no identifiable information on the survey participants; therefore, this work is exempted from any ethical review.

### Outcome Events

‘Adolescence’ in this study refers to teenage motherhood, and adolescent women are defined as ever-married women who have had the experience of childbirth in their teens (aged 15–19) during the three years preceding the date of the respective surveys. The childbearing among women in India is prevalent in wedlock, and since most of the reproductive and child health indicators refer to ever-married women in the reproductive age of 15–49, the NFHS target the sample of ever-married women to canvass the required information. Adolescent maternity care was measured using progress through two vital maternal healthcare indicators: full antenatal care (Full ANC), and professional attendance at delivery (PAD). Full antenatal care includes those women who had had a minimum of three antenatal visits, at least two tetanus toxoid injections during pregnancy or received one tetanus toxoid injection during pregnancy and at least one in the three years prior to the pregnancy, and received iron and folic acid tablets for 90 days or more [18]. Provision of all components of antenatal care to pregnant women is an integral part of the Reproductive and Child Health Program in India. However, in order to maintain consistency across the information available in the three rounds of the NFHS, the full antenatal care indicator was measured as women with at least three antenatal check-ups, and who received at least one tetanus toxoid injection and iron and folic acid tablets or syrup. World Health Organization (WHO) considers delivery conducted either in a medical institution, or home delivery assisted by a doctor/nurse/lady health visitor (LHV)/auxiliary nurse midwife (ANM)/other health professional as ‘safe delivery’ [24]. Following the WHO definition, the PAD is constructed in Indian context, which includes the institutional delivery (i.e. delivery conducted in public or private hospital/health centre/clinic) and deliveries conducted at home with professional assistance by trained health personnel (i.e. doctor/nurse/midwife/auxiliary midwife).

### Covariates

The key socioeconomic predictors of interest, which have prolific scope and influence in government policies and programs include education of women and their husbands, social group, type of residence and economic status. The selection of these variables is based on socioeconomic deprivation factors that persist, and on previous studies on adolescent maternity care [12], [25], [26]. The women’s education indicator was based on information related to the attainment of a particular level of education or no education. This was categorized as: illiterate/no education, literate or below primary, primary, middle, and high school and above. A similar procedure was applied to construct their husband’s education. The social group variable includes the following categories: Scheduled Castes (SC), Scheduled Tribes (ST), and ‘Others’ (or non-SC/ST). This classification used the terminology adopted by the government of India, which focused more on the socially disadvantaged castes/tribes, and all privileged caste groups were represented in the ‘Others’ group. The level of economic status of women was assessed computing a composite index of household wealth indicating possession of wealth or assets by the household to which they belonged. In the absence of direct data on income in household sample surveys like NFHS, the wealth index is widely used as a surrogate indicator for assessing the economic status of the household, and the index has been found to correlate highly with income data in developing countries [27], [28]. In the third round of the NFHS, the wealth index was created using principal component analysis (PCA) on items related to possession of durable assets, access to utilities and infrastructure, and housing characteristics. The PCA scores in the dataset were weighted by the household sampling weights to ensure that the distribution was representative of all the households in India, and then the households were divided into quintiles. The wealth index (quintile) was computed for other rounds of the survey separately using the methodology followed in the third round of NFHS. A detailed description on the methodology adopted to construct the wealth index in NFHS dataset is provided in the NFHS-3 national report [23]. Other social and demographic variables were also used as covariates in the multivariate models, which include mother’s age at childbirth (<18 years, and 18–19 years), religion (Hindu, Muslim, and others), work status (not working, working at home, and working away from home), parity (1, 2, and 3+), birth order and interval (first birth order, birth order 2+ and interval ≤24 months, and birth order 2+ and interval >24 months), mass-media exposure (no exposure, and any exposure), desirable status of the child (wanted, and unwanted), and region of residence (north, central, east, northeast, west, and south).

### Statistical Analyses

In order to trace the trend in the utilization of antenatal care services and professional attendance at delivery among adolescent women, we assessed whether the association between the predictor of interest and the outcome variable varied by survey rounds. This required the data from all three rounds to be pooled and tested for the trend using linear or nonlinear trend analysis. As the sampling design of the NFHS offers an opportunity to make all the three rounds of data comparable [29], several earlier studies have pooled the different rounds of DHS/NFHS datasets to observe changes over time [30]–[32]. Prevalence (%) estimates with 95% confidence intervals (CI) were calculated for adolescent women by subgroups of key socioeconomic indicators using NFHS-calculated individual weights to take into account the multistage sampling design. Cochran-Armitage tests [33] were used to test linear time-trend estimates, while χ^2^ (Chi squared) tests were used for nonlinear trends with tests adjusted for complex survey design [34]. To take into account the survey design (i.e. sampling weights with clustering and strata) while estimating bivariate and multivariate statistics, the SVY command [35] in STATA [36] was used.

Since both the outcome indicators used in this study were measured with binary responses in all respective surveys, we used pooled multivariate logistic regression models to assess the influence or the strength of selected key socioeconomic predictors in explaining adolescents’ maternity care. The models were adjusted for a set of socio-demographic factors. The results of the logit models are presented in terms of odds ratios (OR) with their 95% CI. We additionally fit models stratified by survey periods and with interactions among key socioeconomic predictors to show the extent of disparity in the utilization of healthcare services among adolescents belonging to different socioeconomic strata. The interactions between the predictor of interest and the survey periods in the pooled dataset were analyzed using Wald tests. For unambiguous presentation of the logit models with number of interaction terms, we report the model-based predicted probabilities (PP), along with their 95% CI. Analyses were performed using statistical software, STATA version 10 and Microsoft Excel.

## Results

The proportion of adolescent women availing themselves of full antenatal care (ANC) in India increased by nearly three percentage points from a level of 37% during 1990–93 to 40% during 2003–06 (**Table 1**). However, there was an increase of nearly 14 percentage points (39%) in the overall proportion of adolescent women availing themselves of professional attendance at delivery (PAD) during the same period (**Table 2**). Tables 1 and 2 present the trends in the proportion of adolescent women availing themselves of full ANC and PAD by their socioeconomic background. A considerable growth in the level of use was observed in the middle and the richest wealth quintiles and among adolescent women belonging to the Scheduled Tribes (ST). The prevalence (%) of full ANC among rural adolescent women grew by 7%, with no indication of significant difference in use among urban adolescent women over the period, whereas PAD among rural adolescents grew by 49%, compared to an increase of about 12% among urban adolescents. A considerable increase was observed in the proportion of PAD availed by uneducated adolescents and adolescents whose husbands were uneducated.

**Table 1:** Prevalence (%) of adolescent women receiving full antenatal care by socioeconomic and other select characteristics, India, 1990–2006.

	Full Antenatal Care							
	NFHS 1 (1990–93)		NFHS 2 (1996–99)		NFHS 3 (2003–06)		Relative Change (%)^a^	*p*-value for trend^b^
**Women’s age at childbirth**								
<18	33.6	(30.8–36.5)	28.9	(26.1–31.8)	35.0	(31.0–39.3)	4.1	0.023
18–19	38.9	(36.7–41.1)	36.4	(34.2–38.7)	41.7	(38.9–44.6)	7.3	0.018
**Area of residence**								
Rural	33.8	(31.7–35.9)	30.1	(28.1–32.3)	36.2	(33.5–39.0)	7.2	0.002
Urban	53.1	(48.6–57.5)	52.7	(48.1–57.2)	56.4	(51.2–61.5)	6.4	0.504
**Women’s education**								
Illiterate	26.2	(24.3–28.2)	21.7	(19.6–23.9)	25.7	(22.7–28.9)	–2.0	0.012
Literate or below primary	47.6	(41.7–53.6)	41.2	(35.8–46.8)	43.1	(36.3–50.2)	–9.4	0.320
Primary	53.5	(49.0–57.9)	46.8	(42.3–51.4)	51.0	(46.3–55.6)	–4.8	0.142
Middle	69.2	(63.2–74.6)	55.8	(50.5–61.0)	49.7	(44.2–55.2)	−28.2	<0.001
High school and above	76.4	(69.4–82.3)	67.3	(60.9–73.1)	65.9	(58.8–72.3)	−13.8	<0.001^†^
**Husband’s education**								
Illiterate	25.9	(23.4–28.5)	25.7	(22.8–28.7)	29.1	(25.3–33.2)	12.5	0.292
Literate or below primary	34.4	(29.4–39.7)	37.0	(31.6–42.8)	41.7	(34.8–49.0)	21.5	0.525^†^
Primary	45.6	(41.4–49.9)	31.9	(28.1–36.0)	45.4	(40.5–50.4)	−0.4	<0.001
Middle	42.8	(38.4–47.3)	33.3	(29.4–37.4)	40.1	(35.2–45.1)	−6.4	0.010
High school and above	48.1	(44.1–52.0)	44.2	(40.6–47.9)	47.8	(43.1–52.7)	−0.5	0.335
**Social group**								
SC	35.5	(31.2–39.9)	30.8	(27.3–34.6)	35.2	(30.4–40.2)	–0.8	0.228
ST	22.0	(17.8–26.8)	17.8	(14.1–22.1)	35.7	(29.7–42.3)	62.8	<0.001
Other than SC and ST	39.6	(37.5–41.7)	37.6	(35.2–40.1)	42.7	(39.5–45.9)	7. 8	0.041
**Wealth quintile**								
Poorest	25.4	(22.5–28.6)	21.1	(18.2–24.2)	26.6	(22.8–30.9)	4.8	0.053
Poorer	28.5	(25.4–31.9)	27.4	(24.6–30.5)	31.2	(27.4–35.1)	9.3	0.301
Middle	37.8	(34.2–41.5)	41.2	(37.3–45.1)	47.2	(42.4–52.1)	25.0	0.009^†^
Richer	53.2	(48.6–57.6)	55.0	(50.1–59.8)	54.2	(48.7–59.7)	2.0	0.870
Richest	57.6	(52.9–62.2)	53.3	(47.3–59.2)	70.0	(62.0–77.0)	21.5	0.004
**Total**	37.1	(35.4–38.8)	33.7	(31.7–35.7)	39.9	(37.3–42.5)	7.6	<0.001

NFHS = National Family Health Survey; SC = Scheduled Castes; ST = Scheduled Tribes.

a Calculated as relative change = [(final period %/period 1%)−1].

b Based on Cochran-Armitage time trend analyses^†^(for linear trend) and χ2 analyses (for nonlinear trend) with Rao-Scott adjustments to assess significant trends over time.

Note: Figures in parentheses are 95% Confidence Intervals for the proportions.

**Table 2:** Prevalence (%) of adolescent women experiencing professional attendance at delivery by socioeconomic and other select characteristics, India, 1990–2006.

	Professional Attendance at Delivery							
	NFHS 1 (1990–93)		NFHS 2 (1996–99)		NFHS 3 (2003–06)		Relative Change (%)^a^	*p*-value for trend^b^
**Women’s age at childbirth**								
<18	34.3	(31.3–37.3)	38.1	(35.2–41.1)	43.9	(39.6–48.3)	28.2	<0.001
18–19	36.8	(34.6–39.1)	44.2	(41.8–46.5)	51.9	(49.1–54.7)	41.0	<0.001
**Area of residence**								
Rural	30.1	(28.1–32.2)	36.8	(34.7–39.0)	44.8	(42.1–47.6)	49.0	<0.001
Urban	64.3	(59.7–68.7)	69.3	(64.7–73.6)	71.8	(66.6–76.5)	11.7	<0.001
**Women’s education**								
Illiterate	24.9	(23.0–27.0)	28.3	(26.2–30.5)	33.5	(30.4–36.9)	34.6	<0.001
Literate or below primary	40.8	(35.0–46.9)	50.1	(44.7–55.5)	47.4	(40.2–54.7)	16.0	0.101^†^
Primary	57.2	(52.6–61.7)	58.4	(54.1–62.7)	59.1	(54.4–63.7)	3.3	0.001
Middle	65.2	(58.9–71.1)	66.1	(61.1–70.9)	66.4	(60.7–71.7)	1.9	<0.001
High school and above	76.0	(69.2–81.7)	77.8	(71.6–83.0)	86.3	(80.2–90.7)	13.5	<0.001
**Husband’s education**								
Illiterate	24.4	(21.9–27.2)	29.0	(26.2–32.1)	35.6	(31.6–39.7)	45.7	<0.001
Literate or below primary	35.5	(30.4–40.9)	35.7	(30.7–40.9)	41.5	(34.6–48.8)	17.0	0.214
Primary	40.9	(36.9–45.0)	43.8	(39.7–47.9)	50.8	(45.8–55.7)	24.2	0.895
Middle	43.1	(38.7–47.7)	47.4	(43.2–51.6)	55.0	(50.2–59.7)	27.5	<0.001
High school and above	48.4	(44.7–52.3)	56.2	(52.6–59.7)	65.4	(60.9–69.7)	35.1	0.556
**Social group**								
SC	33.6	(29.4–38.2)	38.0	(34.2–41.9)	45.4	(40.4–50.4)	34.9	<0.001
ST	15.9	(12.6–19.9)	21.6	(17.5–26.5)	38.2	(32.1–44.7)	139.7	<0.001
Other than SC and ST	39.3	(37.1–41.6)	46.9	(44.4–49.3)	54.4	(51.2–57.5)	38.3	<0.001
**Wealth quintile**								
Poorest	23.9	(21.0–27.0)	25.2	(22.0–28.6)	30.9	(27.0–35.1)	29.5	0.084
Poorer	29.2	(26.1–32.5)	35.1	(32.1–38.3)	41.6	(37.4–45.9)	42.7	0.754
Middle	35.5	(32.0–39.1)	49.6	(45.9–53.2)	56.8	(51.4–62.1)	60.2	0.002
Richer	50.9	(46.3–55.4)	67.8	(63.0–72.3)	68.2	(62.7–73.3)	34.2	<0.001
Richest	56.9	(52.2–61.4)	71.7	(66.3–76.5)	90.6	(85.7–94.0)	59.3	<0.001
**Total**	35.9	(34.0–37.9)	42.0	(39.9–44.0)	49.8	(47.2–52.3)	38.5	<0.001

NFHS = National Family Health Survey; SC = Scheduled Castes; ST = Scheduled Tribes.

a Calculated as relative change = [(final period %/period 1%)−1].

b Based on Cochran-Armitage time trend analyses (for linear trend) and χ2 analyses^†^ (for nonlinear trend) with Rao-Scott adjustments to assess significant trends over time.

Note: Figures in parentheses are 95% Confidence Intervals for the proportions.

**Table 3** presents the influence of socioeconomic predictors on the use of select maternity healthcare services by adolescents during 1990–2006, controlling for a set of socio-demographic and regional factors. The overall probability of adolescents availing themselves of full ANC appeared to increase by only 4% between 1990–93 and 2003–06 (although the OR does not indicate to be with 95% statistical confidence), whereas the probability of adolescents availing themselves of PAD grew by 79% during the same period. The probability increased significantly with the increasing economic level (that is, wealth quintile) of the users. Women’s education transpired as the most influential socioeconomic predictor leading to higher probability of adolescents availing themselves of full ANC and PAD during 1990–2006. However, the husband’s education level had a non-linear but positive influence on the outcome. Adolescent women belonging to the ST social group appeared to be disadvantaged with 25% less probability to avail themselves of full ANC compared to non-SC/ST adolescents, whereas rural adolescents were also almost equally disadvantaged (with 25% less probability) than their urban counterparts. Similarly, SC and ST adolescents had 13% and 39% less probability to avail themselves of PAD compared to non-SC/ST adolescents, respectively. The rural adolescents were more than 2 times less probable to avail themselves of PAD compared to their urban counterparts.

**Table 3:** Selected socioeconomic predictors for adolescent women accessing full antenatal care and professional attendance at delivery, India, 1990–2006.

Adolescent women (N = 12164)	Full Antenatal Care^a^		Professional Attendance at Delivery^b^	
	OR	(95% CI)	OR	(95% CI)
**Period**				
1990–93	1		1	
1996–99	0.88	(0.77–1.00)	1.53	(1.34–1.74)
2003–06	1.04	(0.89–1.21)	1.79	(1.55–2.08)
	*p* = 0.075		*p*<0.001	
**Wealth quintile**				
Poorest	1		1	
Poorer	1.08	(0.93–1.26)	1.18	(1.01–1.37)
Middle	1.29	(1.10–1.53)	1.21	(1.02–1.43)
Richer	1.57	(1.28–1.91)	1.45	(1.19–1.77)
Richest	1.79	(1.44–2.22)	1.60	(1.28–1.99)
	*p*<0.001		*p*<0.001	
**Women’s education**				
Illiterate	1		1	
Literate or below primary	1.87	(1.57–2.24)	1.48	(1.24–1.77)
Primary	1.98	(1.70–2.30)	1.83	(1.58–2.12)
Middle	2.60	(2.16–3.14)	2.14	(1.76–2.59)
High school and above	3.05	(2.38–3.91)	2.80	(2.14–3.65)
	*p*<0.001		*p*<0.001	
**Husband’s education**				
Illiterate	1		1	
Literate or below primary	1.27	(1.05–1.54)	1.02	(0.83–1.25)
Primary	1.29	(1.10–1.50)	1.15	(0.98–1.34)
Middle	1.21	(1.03–1.43)	1.36	(1.17–1.60)
High school and above	1.17	(0.99–1.37)	1.31	(1.11–1.54)
	*p* = 0.011		*p*<0.001	
**Social group**				
Other than SC and ST	1		1	
SC	0.95	(0.83–1.10)	0.87	(0.76–0.99)
ST	0.75	(0.62–0.91)	0.61	(0.51–0.75)
	*p* = 0.015		*p*<0.001	
**Area of residence**				
Rural	1		1	
Urban	1.26	(1.09–1.45)	2.19	(1.87–2.56)
	*p* = 0.002		*p*<0.001	

SC = Scheduled Castes; ST = Scheduled Tribes.

a Multivariate model controls for mother’s age at childbirth, religion, work status, parity, birth order & interval, mass-media exposure, desirable status of the child, and region besides all variables shown in the table.

b Model controls for utilization of full antenatal care besides all variables mentioned above.

Note: *p*-value refers to adjusted Wald test.

However, when these individual factors integrate at the stark opposite end of the socioeconomic spectrum, the situation becomes unappreciable and intimidating from the policy and program point of view. Rural adolescent women with no education in the poorest wealth quintile were almost 55–60 percentage points and 70–72 percentage points less likely to avail full ANC and PAD, respectively, compared to urban adolescents with high school education and more in the richest wealth quintile during 1990–2006 (**Table 4**). Interacting with the ethnic affiliation of adolescents, the probability of availing full ANC and PAD by rural SC/ST adolescents belonging to the poorest wealth quintile was 43–50 percentage points and 62–64 percentage points lower compared to urban non-SC/ST adolescents in the richest wealth quintile, respectively. Similarly, the rural SC/ST adolescent women with no education in the poorest wealth quintile had 63–65 percentage points and 72–74 percentage points less probability to avail themselves of full ANC and PAD, respectively, compared to urban SC/ST adolescents with high school education and more in the richest wealth quintile over the period.

**Table 4:** Adjusted predicted probability (PP) for paired interactions of socioeconomic characteristics of adolescent women accessing full antenatal care and professional attendance at delivery, India, 1990–2006.

	NFHS- 1 (1990–93)		NFHS-2 (1996–99)			NFHS-3 (2003–06)		
*Paired Interactions of Covariates*	PP	(95% CI)	PP	(95% CI)		PP		(95% CI)
*Full Antenatal Care* 1								
Rural*No education*Q1^a^	0.173	(0.155–0.193)	0.138	(0.121–0.158)		0.162		(0.140–0.188)
Urban*HS & above*Q5^b^	0.746	(0.696–0.790)	0.694	(0.638–0.746)		0.766		(0.715–0.810)
Rural*SC/ST*Q1^c^	0.172	(0.151–0.194)	0.157	(0.137–0.180)		0.201		(0.174–0.231)
Urban*Non SC/ST*Q5^d^	0.614	(0.570–0.657)	0.591	(0.542–0.638)		0.698		(0.652–0.742)
Rural*SC/ST*No education*Q1^e^	0.150	(0.137–0.165)	0.135	(0.124–0.147)		0.150		(0.136–0.165)
Urban*Non SC/ST*HS & above*Q5^f^	0.783	(0.756–0.806)	0.763	(0.739–0.784)		0.800		(0.778–0.821)
*Professional Attendance at Delivery* 2								
Rural*No education*Q1	0.145	(0.128–0.163)	0.174	(0.155–0.196)		0.216		(0.191–0.243)
Urban*HS & above*Q5	0.864	(0.830–0.893)	0.893	(0.865–0.917)		0.920		(0.897–0.938)
Rural*SC/ST*Q1	0.134	(0.117–0.153)	0.184	(0.162–0.208)		0.252		(0.223–0.282)
Urban*Non SC/ST*Q5	0.753	(0.714–0.788)	0.826	(0.794–0.853)		0.885		(0.860–0.906)
Rural*SC/ST*No education*Q1	0.130	(0.118–0.142)	0.148	(0.136–0.161)		0.209		(0.191–0.227)
Urban*Non SC/ST*HS & above*Q5	0.867	(0.849–0.883)	0.886	(0.871–0.899)		0.925		(0.913–0.935)
*Proportion of adolescent women*	%	(95% CI)	%	(95% CI)		%		(95% CI)
Rural*No education*Q1	36.1	(32.3–40.2)	38.7	(34.9–42.7)	25.1		(22.2–28.3)	
Urban*HS & above*Q5	31.6	(23.4–41.1)	32.8	(23.9–43.1)	35.6		(27.2–45.1)	
Rural*SC/ST*Q1	26.7	(22.7–31.2)	39.1	(34.7–43.7)	34.2		(30.0–38.6)	
Urban*Non SC/ST*Q5	41.1	(34.7–47.8)	34.9	(28.9–41.4)	24.0		(19.1–29.8)	
Rural*SC/ST*No education*Q1	29.6	(25.2–34.5)	39.7	(35.1–44.6)	30.6		(26.3–35.4)	
Urban*Non SC/ST*HS & above*Q5	35.3	(25.9–46.0)	35.9	(25.8–47.4)	28.8		(20.6–38.7)	

NFHS = National Family Health Survey; SC = Scheduled Castes; ST = Scheduled Tribes.

1 Multivariate model controls for mother’s age at childbirth, religion, work status, parity, birth order & interval, mass-media exposure, desirable status of the child, and region besides all variables shown in the table.

2 Model controls for utilization of full antenatal care besides all variables mentioned above.

a Rural adolescent women with no education in the poorest wealth quintile.

b Urban adolescent women with high school & above education in the richest wealth quintile.

c Rural SC/ST adolescent women in the poorest wealth quintile.

d Urban non-SC/ST adolescent women in the richest wealth quintile.

e Rural SC/ST adolescent women with no education in the poorest wealth quintile.

f Urban non-SC/ST adolescent women with high school & above education in the richest wealth quintile.

Note: All the predicted probabilities were significantly different at *p*<0.001.

## Discussion

The present study has comprehensively demonstrated the trends in adolescent women’s availing themselves of full ANC and PAD with considerable disparities across key socioeconomic spectrum in India during 1990–2006. Over the last one and a half decades, a negligible and modest increase in receiving full antenatal care and professional attendance at delivery, respectively, was evident among adolescent women. The prime catch of this entire exploration remained concentrated on years-long persistence of desolate disparities in use of healthcare services across socioeconomic strata, which clearly suggests low priority in addressing social determinants of health among adolescents over the period. The difference was almost two to three fold between the lower and upper structures of the educational, economic and social spectrum. A range of published literature highlighting the key socioeconomic factors affecting the utilization of maternal healthcare services in India is available, which reiterates the influence of place of residence [37], education [13], wealth [38] and social groups [39] on the use of maternity care services. However, few studies have demonstrated the extent of disparities between the least and the most favoured groups across the socioeconomic spectrum. This study comes across a manifestation of huge disparities between adolescents of the least and the most favoured groups in availing themselves of full antenatal care and skilled delivery care in India. Besides, this study also estimates a considerable proportion of adolescents in the least favoured group, which is more than one-fourth of the total adolescent women in India, and thus, they cannot be ignored.

Irrespective of world regions, global literature has shown substantial differences in the utilization of maternity healthcare services between urban and rural areas [40]. The rural disadvantage in healthcare utilization has often been attributed to the substandard modern healthcare system [41], along with lack of basic civic amenities and facilities [42]. According to Rural Health Statistics –2010, there was a shortage of about 19590 sub-centres, 4252 PHCs and 2115 CHCs in India [43], which has serious implications for ensuring timely and appropriate healthcare services delivery among adolescent women who are in need. Evidences from India [44] and elsewhere [45], [46] have revealed education as one of the most effective and proven strategies to maximize the utilization of maternal healthcare services. This finding is relevant for adolescent women, as nearly two in five adolescent women in the study sample did not complete their primary level of education during 2003–06. Despite extensive governmental and non-governmental efforts to delay the age of marriage, about 43% of currently married women aged 20–24 reported to be married before the legal age at marriage of 18 years in 2010, and it ranges from the highest in Bihar (68%) to the lowest in Himachal Pradesh (9%) [47]. The early marriages of girls are evidenced and contemplated as a hazard for the intellectual and physical growth of mothers. Early marriages foster incidences of early school dropouts among adolescents and their engagement in household chores, especially in rural settings [11]. Being the newlywed bride, their movement outside home at most of the occasions is restricted, justifying the concern as social or cultural norms. Thus, the adolescents’ low level of education or no education coupled with the issues of full-time engagement in domestic work and social restrictions on their movement tend to compel them having low mass-media exposure and awareness towards availability and necessity of healthcare services at particular occasions.

Recent evidence from India has demonstrated increasing inequality in the utilization of maternal healthcare services across economic groups [38]. It has been argued that the low coverage of maternal healthcare services among poor households could be the outcome of their priority to meet basic daily needs, rather than spend their limited resources on healthcare [48]. In addition, policies and programs designed for the poor are more likely to end up as ‘poor programs’, and fail to achieve their desired objective [49]. The proportion of adolescent married women belonging to the poorest and poorer wealth quintiles in India increased from 50% in 1992 to over 60% in 2005, suggesting an urgent policy response to target poor adolescent women to meet their safe reproductive needs. Moreover, the long history of social and spatial segregation of people into different social strata along with their cultural practices has been the leading cause of underutilization of healthcare services among certain sections of population. Despite decades of affirmative action allocated to the constitutionally scheduled social groups by the government of India, the outcome does not appear to be effective.

The overall trend that attracts the attention is the manifestation of the difference of almost ten percentage points in the proportional utilization of full antenatal care and professional delivery care among adolescents during 1996–99 and 2003–06, while the proportion of adolescents receiving either of these maternity care services were almost similar during 1990–93. Although, even during 2003–06, the proportion of adolescents receiving professional delivery care was not satisfactory, as half of the adolescents did not receive professional attendance during their childbirth. Nonetheless, the trend suggests a higher growth in the utilization of professional delivery care services compared to the full antenatal care services among adolescents, which is also validated by the result of multivariate analyses. This trend implies certain underlying facts. First, adolescent women or their husbands or families do not understand the importance of antenatal care services. Second, the social barriers for the newlywed brides or the economic constraints compel them to compromise with a few healthcare services, and thus, the family or the mother decides to expend on highly required services. Third, the quality of antenatal care services provided by most of the public hospitals/sub-centers might discourage their frequent visits to healthcare centers. Additionally, evidences suggest that a substantial proportion of young women reported the experience of at least one pregnancy-related complication during pregnancy for the first birth [50] that could lead to adolescents being more attentive towards receiving professional attendance during delivery.

Although, with effective reproductive health interventions through international and national sponsored programmes, the progress in availing professional delivery care among adolescents is evident from the analysis, however, the stagnated proportion of utilization of full ANC over the period is real concern. Without having appropriate knowledge and prerequisite measures and precautions during the gestation period, the adolescents may be at risk even while having professional or skilled attendance at delivery. This has profound implications on reproductive outcomes. In absence of appropriate antenatal consultation and practicing recommended precautions and medications, there are chances of threat to both mother and child during delivery, which may be resulted into miscarriage/stillbirth or any abnormality in newborn. On the other hand, receiving appropriate antenatal consultation and care can enhance the chances of adolescents getting professional or skilled attendance at delivery [12], [13] and postnatal care [51]. This study manifests that the overall utilization of full antenatal care services among adolescents can be increased if they get educated and if the adolescents belonging to SC/ST families and in rural areas could get economic incentives to avail this important reproductive healthcare service during pregnancy. The economic constraints also appeared to be a pertinent factor of low utilization of full ANC among adolescents. Consequently, the effective antenatal consultation will follow the better utilization of other reproductive and child healthcare services.

### Policy Implications

The issue addressed in this paper on adolescents’ maternity care has important implications for the national health policy. The need for improved maternal health has long been an integral part of India’s contemporary health policy, particularly since the integration of the Child Survival and Safe Motherhood (CSSM) program under the broad umbrella of the Reproductive and Child Health Program (RCH) in 1997. However, no specific attempts have been made to acknowledge adolescents’ maternity healthcare needs. The health program initiatives typically grouped all married women regardless of their residential segregation, age at marriage and socioeconomic characteristics –that masks the lower coverage of maternity health benefits among certain groups (for instance, adolescent women) over the average achievement. This ‘missing link’ between programmes and poor adolescents’ maternal healthcare over the last one and a half decades could only be addressed with specific policies and schemes that acknowledge the needs of adolescents and newlywed young women.

Since 2005, the government of India has launched several programs, including the National Rural Health Mission (NRHM), *Navjaat Shishu Suraksha Karyakram* (NSSK) towards strengthening health facilities, particularly in rural areas. It is evident from this paper that without addressing the social determinants of health, highlighted by the WHO Commission on Social Determinants of Health [52] and the Rio political declaration, 2011 [53], adolescent women would remain disadvantaged in maternity healthcare benefits. With such academic authority, the present study recommends focused initiatives under the ongoing programmatic efforts to reach out to the most vulnerable adolescents who are in need of essential maternal healthcare services. The importance of addressing early marriage and childbearing pattern cannot be ignored. Recent estimates suggest that 25% of adolescent women have reported unmet need of family planning (birth spacing) services [47] that need to be addressed effectively to avert unplanned pregnancies. In this regard, local health workers have a vital role towards providing essential counseling to eligible couples. In 2006, the government of India passed the Prohibition of Child Marriage Act-2006 [54], which restricts minimum age at marriage to 21 years for boys and 18 years for girls. This study reemphasizes serious efforts to enforce the law with the help of various stakeholders, and make local bodies such as *panchayats* (in rural areas) accountable for its effective implementation [13].

Recently, the Planning Commission, government of India instituted a High Level Expert Group (HLEG) in order to improve universal health coverage in India [55]. The group has listed certain high priority healthcare action to be undertaken that includes the expansion of primary healthcare, strengthening of district hospitals, expansion and upgrading the skills of the health workforce, free provision of essential medicines, abolition of ‘user fees’, establishment of effective regulatory structures and support for active community participation, which require early implementation. In addition, the HLEG suggested the ‘National Health Package’ for essential health at the primary, secondary, as well as tertiary levels of care for all citizens of India by 2022. The present study suggests the promotion of a comprehensive ‘adolescent scheme’ along the lines of ‘Continuum of Maternal, Newborn and Child health Care’ to address the unmet need of reproductive and maternal healthcare services among adolescent women in India.

### Limitations of this Study

A few limitations of this study are to be acknowledged too. Although, the childbearing among women in India is largely practiced in wedlock, the result of this study cannot be generalized for all adolescent women, as the sample in the analysis included only those adolescents who had ever been married. The population belonging to the so-called “Other Backward Classes” (OBC) are identified as economically and socially deprived by the Mandal Commission that reported to the Indian government in 1980 [56]. Hence, this becomes important to distinguish and/or compare OBC from SC, ST, and the privileged “Other” social/caste group, whenever an effort is made to assess the developmental aspects of the Indian society. Since the NFHS-1 (1992–93) data contained only three categories of the social/caste group –SC, ST and Other, we were forced to pool OBC and the Other social group in the successive two NFHS rounds (i.e., NFHS-2 (1998–99), and NFHS-3 (2005–06)), which provided separate information for OBC. This study may include recall errors or social desirability bias while reporting information on the utilization of maternity care services, as usually referred in the case of survey dataset. The socioeconomic and demographic indicators selected for this study, though pertinent, do not cover issues like the quality of healthcare services available, accessibility, or as a whole, the quality of governance, which varies widely across states, and with successive governments and state administrations within states.

### Conclusion

While the global and the national focus are on achieving MDG 5 targets by reducing maternal mortality (MMRs) through adoption of utilitarian strategies for the utilization of maternity care services, this has been assessed in many low-income and developing countries that the goal is unlikely to be achieved until the services reach to the marginalized. The marginalization of adolescents in utilizing maternity care services in India over one and a half decades is clearly manifested in this study. In addition, the socioeconomic vulnerability among considerable population of adolescent women, who are disadvantaged in using maternity care services, could foster adverse reproductive outcomes. This has immense implication in the regulation of maternal mortality in India.

The unfortunate reproductive incidences among adolescents can be prevented by prohibiting the marriage at early age, and if married, by delaying the childbearing or the effective provision of maternity care services. Girl’s education is the most important weapon to deal with such concerns in any society. Besides, mobilizing parents and community members towards the importance of specific healthcare services and its after effects can be an effective strategy. The role of local non-governmental organizations (NGOs) and young women’s advocacy groups at the community level may prove effective in raising awareness about the consequences of young women’s early marriages and the evasion of healthcare services during pregnancy. Offering economic support and incentives to girls and their families, especially to girls belonging to the least advantaged groups in socioeconomic spectrum in rural areas can also be one of the effective ways of preventing early childbearing. The rationale behind this approach is that immediate economic opportunities provide an acceptable alternative to marriage, and increase the value and contribution of a daughter to her parental family. However, the immediate response to the vulnerability of adolescents in availing required healthcare services is to extend the subsidized maternity healthcare services to adolescent women as well.

## Supporting Information

Appendix S1 **Analytic sample used for analyses from three NFHS rounds by selected variables.**
(DOC)
