# Supplementary material for: Socioeconomic Disparities in Maternity Care among Indian Adolescents, 1990–2006
Source: PLoS One. 2013 Jul 23;8(7):e69094. doi: 10.1371/journal.pone.0069094 (PMC3720871; doi:10.1371/journal.pone.0069094)
Supplement: Appendix S1 — Analytic sample used for analyses from three NFHS rounds by selected variables. (DOC) [file pone.0069094.s001.doc]

**Appendix S1.** Analytic sample used for analyses from three NFHS rounds (adolescent women in last three years preceding the date of respective surveys) by selected variables

| ***Socioeconomic and demographic variables*** | NFHS 1  (1990-93) | |  | NFHS 2  (1996-99) | |  | NFHS 3  (2003-06) | | *p*-value |
| --- | --- | --- | --- | --- | --- | --- | --- | --- | --- |
|  | % | n |  | % | n |  | % | n |  |
| **Women's age at childbirth** |  |  |  |  |  |  |  |  | <0.001 |
| <18 | 34.4 | 1,528 |  | 36.2 | 1,527 |  | 27.0 | 764 |  |
| 18-19 | 65.6 | 3,137 |  | 63.8 | 2,938 |  | 73.0 | 2,270 |  |
| **Area of residence** |  |  |  |  |  |  |  |  | 0.516 |
| Rural | 82.9 | 3,750 |  | 84.2 | 3,653 |  | 81.8 | 2,150 |  |
| Urban | 17.1 | 915 |  | 15.8 | 812 |  | 18.2 | 884 |  |
| **Women's education** |  |  |  |  |  |  |  |  | <0.001 |
| Illiterate | 66.6 | 3,008 |  | 58.3 | 2,542 |  | 45.2 | 1,238 |  |
| Literate or below primary | 7.5 | 358 |  | 10.2 | 448 |  | 9.1 | 304 |  |
| Primary | 14.2 | 688 |  | 16.0 | 736 |  | 23.7 | 714 |  |
| Middle | 7.1 | 375 |  | 9.5 | 462 |  | 13.6 | 490 |  |
| High school and above | 4.7 | 236 |  | 6.0 | 277 |  | 8.4 | 288 |  |
| **Husband’s education** |  |  |  |  |  |  |  |  | <0.001 |
| Illiterate | 38.5 | 1,729 |  | 31.6 | 1,347 |  | 29.0 | 830 |  |
| Literate or below primary | 9.6 | 427 |  | 10.0 | 446 |  | 9.2 | 280 |  |
| Primary | 16.9 | 800 |  | 17.4 | 796 |  | 19.1 | 600 |  |
| Middle | 13.7 | 688 |  | 16.5 | 793 |  | 19.3 | 610 |  |
| High school and above | 21.2 | 1,021 |  | 24.5 | 1,083 |  | 23.4 | 714 |  |
| **Social group** |  |  |  |  |  |  |  |  | <0.001 |
| SC | 13.8 | 652 |  | 21.9 | 929 |  | 25.2 | 664 |  |
| ST | 11.1 | 649 |  | 10.9 | 646 |  | 11.4 | 483 |  |
| Other than SC and ST | 75.1 | 3,364 |  | 67.3 | 2,850 |  | 63.4 | 1,743 |  |
| **Wealth quintile** |  |  |  |  |  |  |  |  | <0.001 |
| Poorest | 25.5 | 1,095 |  | 28.3 | 1,125 |  | 26.3 | 666 |  |
| Poorer | 25.7 | 1,141 |  | 31.5 | 1,323 |  | 28.1 | 784 |  |
| Middle | 21.5 | 1,020 |  | 20.8 | 987 |  | 22.8 | 735 |  |
| Richer | 13.3 | 706 |  | 11.7 | 609 |  | 16.3 | 578 |  |
| Richest | 14.0 | 703 |  | 7.7 | 421 |  | 6.4 | 271 |  |
| **Religion** |  |  |  |  |  |  |  |  | 0.456 |
| Hindu | 81.4 | 3,673 |  | 80.3 | 3,469 |  | 81.3 | 2,250 |  |
| Muslim | 14.9 | 667 |  | 16.6 | 724 |  | 15.7 | 513 |  |
| Others | 3.7 | 325 |  | 3.1 | 272 |  | 3.0 | 271 |  |
| **Working status** |  |  |  |  |  |  |  |  | <0.001 |
| Not working | 72.5 | 3,362 |  | 68.8 | 3,137 |  | 76.0 | 2,333 |  |
| Working at home | 3.7 | 176 |  | 4.5 | 212 |  | 5.4 | 163 |  |
| Working away from home | 23.9 | 1,127 |  | 26.7 | 1,116 |  | 18.5 | 538 |  |
| **Mass-media exposure** |  |  |  |  |  |  |  |  | <0.001 |
| No exposure | 51.0 | 2,328 |  | 48.2 | 2,122 |  | 33.9 | 867 |  |
| Any exposure | 49.0 | 2,337 |  | 51.9 | 2,343 |  | 66.2 | 2,167 |  |
| **Parity** |  |  |  |  |  |  |  |  | 0.007 |
| 1 | 72.4 | 3,448 |  | 70.9 | 3,058 |  | 75.3 | 2,306 |  |
| 2 | 23.5 | 1,042 |  | 24.9 | 1,026 |  | 21.6 | 634 |  |
| 3+ | 4.1 | 175 |  | 4.2 | 164 |  | 3.1 | 94 |  |
| **Birth order & interval** |  |  |  |  |  |  |  |  | 0.012 |
| Birth order 1 | 67.6 | 3,225 |  | 66.5 | 3,024 |  | 71.0 | 2,192 |  |
| Birth order 2+; interval <=24 months | 14.5 | 658 |  | 15.4 | 682 |  | 13.5 | 414 |  |
| Birth order 2+; interval >24 months | 17.9 | 782 |  | 18.2 | 759 |  | 15.5 | 428 |  |
| **Sex of the child** |  |  |  |  |  |  |  |  | 0.581 |
| Female | 48.9 | 2,299 |  | 47.7 | 2,102 |  | 47.9 | 1,488 |  |
| Male | 51.1 | 2,366 |  | 52.3 | 2,363 |  | 52.1 | 1,546 |  |
| **Status of the child** |  |  |  |  |  |  |  |  | 0.002 |
| Wanted | 82.6 | 3,838 |  | 85.8 | 3,827 |  | 85.6 | 2,562 |  |
| Unwanted | 17.4 | 827 |  | 14.2 | 631 |  | 14.4 | 472 |  |
| **Region** |  |  |  |  |  |  |  |  | <0.001 |
| North | 9.2 | 803 |  | 8.1 | 703 |  | 8.4 | 335 |  |
| Central | 23.5 | 1,014 |  | 29.9 | 1,176 |  | 24.9 | 683 |  |
| East | 23.5 | 917 |  | 22.6 | 878 |  | 33.8 | 722 |  |
| North-east | 4.2 | 467 |  | 3.5 | 479 |  | 3.8 | 480 |  |
| West | 15.9 | 508 |  | 13.8 | 448 |  | 12.0 | 307 |  |
| South | 23.8 | 956 |  | 22.2 | 781 |  | 17.2 | 507 |  |

Note: *p*-value represents Chi-squared test for differences in proportions by year group

‘n’ represents unweighted sample size.
